# Impact of systemic immune-inflammation index and systemic inflammation response index on all-cause and cause-specific mortality: a community-based cohort study

**DOI:** 10.3389/fmed.2026.1784058

**Published:** 2026-03-25

**Authors:** Juzhong Ke, Kang Wu, Xiaolin Liu, Hua Qiu, Qingping Liu, Jiahui Song, Yang Liu, Xiaonan Ruan, Yi Zhou

**Affiliations:** Shanghai Pudong New Area Center for Disease Control and Prevention (Shanghai Pudong New Area Health Supervision Institute), Shanghai, China

**Keywords:** cohort study [or longitudinal study], epidemioiogy, mortality, systemic immune-inflammation index (SII), systemic inflammation response index (SIRI)

## Abstract

**Objective:**

To examine the associations of the systemic immune-inflammation index (SII) and systemic inflammation response index (SIRI) with mortality in a Chinese community-based population.

**Methods:**

We analyzed data from 9,318 participants in a community-based prospective cohort Study in Pudong New Area, Shanghai, China. Associations between SII/SIRI and mortality were evaluated using Cox and Fine-Gray models. Non-linear relationships were examined using restricted cubic splines. Stratified analyses and measures of model discrimination and reclassification were also performed.

**Results:**

After multivariable adjustment, the highest SII quartile (Q4) was associated with higher risks of all-cause mortality (HR = 1.35, 95% CI: 1.14–1.61), cardiovascular mortality (HR = 1.33, 95% CI: 1.02–1.73), and respiratory mortality (HR = 3.37, 95% CI: 1.44–7.90), but not cancer mortality. For SIRI, Q4 was associated with higher risks of all-cause mortality (HR = 1.68, 95% CI: 1.39–2.04), cardiovascular mortality (HR = 1.40, 95% CI: 1.05–1.87), cancer mortality (HR = 1.45, 95% CI: 1.02–2.05), and respiratory mortality (HR = 3.07, 95% CI: 1.34–7.02). Significant dose-response relationships were observed for both SII and SIRI with all-cause and cause-specific mortality. Subgroup analysis indicated stronger associations of SIRI with all-cause mortality in participants aged < 60 years. Adding SIRI or SII to conventional risk models improved predictive performance for mortality, with SIRI providing more consistent enhancement across outcomes.

**Conclusions:**

Our findings identify SII and SIRI as independent risk factors for mortality, with SIRI demonstrates superior prognostic value for both all-cause and cause-specific mortality.

## Introduction

While acute inflammation serves as an essential protective response to injury and infection, its chronic, systemic dysregulation becomes profoundly harmful. In adults, persistent low-grade inflammation is recognized as a hallmark of aging. This state transforms into a key pathogenic contributor to several major diseases, including cardiovascular disease, metabolic disorders, and cancer—the leading causes of global disability and mortality (1–3). Elevated levels of inflammatory biomarkers have been consistently shown to independently predict all-cause mortality regardless of other established risk factors (4). Moreover, chronic low-grade systemic inflammation may also mediate the links between environmental exposure and increased risk of all-cause mortality and cardiovascular mortality (5, 6). Global population aging will therefore significantly increase the burden of inflammation-related mortality. Consequently, identifying reliable inflammatory biomarkers to refine mortality risk stratification has become a pressing public health priority.

The systemic immune inflammation index (SII) and systemic inflammation response index (SIRI) are emerging biomarkers that combine three white blood cell subsets with platelets, representing an integrated measure of thrombo-inflammatory and immune status (7, 8). Compared with biomarkers like platelet to lymphocyte ratio, neutrophil to lymphocyte ratio, and C-reactive protein, SII and SIRI are less susceptible to fluctuations caused by dehydration or fluid overload than individual blood cell counts (9, 10). These indices reflect both local and systemic inflammatory processes and have been associated with a range of health outcomes, including diabetic depression, pneumonia occurrence, and cancer prognosis (11–13). While previous studies have established associations between SII/SIRI and mortality in Western populations, evidence from large-scale community-based Asian cohorts remains limited. Using data from a community-based cohort in Shanghai's Pudong New Area—a region marked by advanced economic development and one of the highest levels of population aging in China—this study aims to investigate the associations of SII/SIRI with all-cause and cause-specific mortality and to evaluate their prognostic performance. Findings from this distinctive demographic setting are expected to provide timely and relevant insights for other rapidly aging regions, thereby enhancing the public health relevance of existing evidence.

## Materials and methods

### Study population

The study population was derived from an ongoing community-based prospective cohort in Pudong New Area, Shanghai, China, the design of which has been detailed previously (14). Participants were initially enrolled between January and July 2013 using a multistage stratified random cluster sampling method, and written informed consent was obtained from all participants at the time of enrollment. After excluding individuals with type I diabetes and pregnant women, 10,657 eligible individuals (from an initial pool of 12,382) were enrolled at baseline. A total of 1,339 participants were further excluded due to missing blood routine test data (*n* = 130), missing covariate information (*n* = 976), or non-local household registration that precluded reliable follow-up (*n* = 233), resulting in a final analytical sample of 9,318 participants (Supplemental Figure 1). The current study represents a retrospective analysis of this existing dataset. Ethical approval for the current study was granted by the Ethical Committee of Shanghai Pudong New Area Center for Disease Control and Prevention (Shanghai Pudong New Area Health Supervision Institute) (PDCDCLL-20250508-003). All methods were carried out in accordance with relevant guidelines and regulations.

### Exposure measurements

All participants fasted overnight and withheld hypoglycemic medications for 12 h. Fasting blood samples were then collected from the antecubital vein for laboratory analysis, followed by an oral glucose tolerance test (OGTT). Complete blood count, glucose, and lipids were measured on a HITACHI 7170A automated analyzer. The SII was calculated using the formula: (platelet count × neutrophil count)/lymphocyte count, while the SIRI was calculated using the formula: (neutrophil count × monocyte count)/lymphocyte count.

### Covariates

Demographic, lifestyle, and medical history data were collected using a structured questionnaire. Trained investigators from community health centers conducted all interviews face-to-face. We defined current smoking as smoking at least one cigarette per day over the preceding 6 months; alcohol consumption as consuming at least three times per week over the past 6 months; and physical activity as engagement in athletic activities at least once per week over the past 5 years. Hypertension was defined as measured blood pressure ≥140/90 mmHg or use of blood pressure-lowering medication. Type 2 diabetes was defined as fasting plasma glucose ≥7.0 mmol/L, 2h plasma glucose ≥11.1 mmol/L by OGTT, or use of glucose-lowering medication. According to the U.S. NCEP-ATP III criteria, dyslipidemia was defined as plasma triglyceride (TG) ≥2.26 mmol/L, total cholesterol (TC) ≥6.20 mmol/L, low-density lipoprotein cholesterol (LDL) ≥4.13 mmol/L, high-density lipoprotein cholesterol (HDL) < 1.03 mmol/L, or use of cholesterol-lowering medication. Body mass index (BMI) was calculated as weight (kg)/height (m^2^).

### Outcome ascertainment

The primary outcomes were all-cause and cause-specific mortality. Participants were followed from the baseline survey until death or December 31, 2023, whichever came first. Mortality status was ascertained through linkage with the mortality surveillance registry of Pudong New Area, Shanghai, China. Underlying causes of death were classified according to the International Classification of Diseases, 10th Revision (ICD-10), as follows: cardiovascular mortality (I00–I99), cancer mortality (C00–D48), and respiratory mortality (J00–J99).

### Statistical analysis

The primary aim was to assess the association of SII/SIRI with all-cause mortality, while analyses of cause-specific mortality and subgroup explorations are presented as exploratory. Participants were categorized into quartiles according to SII/SIRI levels. Continuous variables are expressed as mean (SD) or median (IQR) and were compared using one-way ANOVA or the Kruskal-Wallis test. Categorical variables are presented as number (%) and were compared using Pearson's chi-square test. The association between SII/SIRI (both as continuous variables and in quartile categories) and all-cause mortality was assessed using Cox proportional hazards models, adjusted for age, sex, residential area, marriage status, education, current smoking, alcohol consumption, physical activity, BMI, hypertension, type 2 diabetes, and dyslipidemia. Collinearity diagnostics and Schoenfeld residual analyses were conducted to validate model robustness and confirm adherence to proportional hazards assumptions in Cox regressions. To account for competing risks, the association between SII/SIRI and cause-specific mortality was evaluated using Fine-Gray proportional sub-distribution hazards models. Regarding the relatively small number of respiratory deaths (*n* = 53), the analyses for respiratory mortality are presented based on models adjusted for age and sex only. Restricted cubic splines were incorporated into Cox models to examine potential non-linear relationships between SII/SIRI (as continuous variables) and mortality, using 3 knots placed at the 5th, 50th, and 95th percentiles of SII/SIRI, respectively, with the median level of SII/SIRI as the reference value. Stratified analyses were conducted to evaluate the association between SII/SIRI and mortality across subgroups defined by age, sex, BMI, hypertension, and diabetes, with the adjusted model excluding the stratification factor. To examine whether SII/SIRI improves the predictive performance of mortality prediction models, discrimination and reclassification were measured by change in C-index, integrated discrimination improvement (IDI) and net reclassification improvement (NRI). The 95% CIs for these measures were estimated using bootstrap. Statistical analyses were performed using SPSS 22.0 and SAS 9.4. A two-sided *p* value of < 0.05 was considered significant.

## Results

### Baseline characteristics

Baseline characteristics of the study participants by SII/SIRI quartiles are presented in Table 1. Of the 9,318 included participants (mean age 57.89 years, 37.86% male), higher levels of SII and SIRI were significantly associated with older age, elevated BMI, lower proportion of married individuals, current smoking, and hypertension. In terms of laboratory parameters, elevated SII and SIRI were associated with unfavorable glycemic and lipid profiles, as well as increased counts of white blood cells, neutrophils, platelets, and monocytes, accompanied by decreased lymphocyte counts. Moreover, higher SIRI was also significantly associated with alcohol consumption, and type 2 diabetes. No consistent trends were observed for sex or physical activity across the groups.

**Table 1:** Baseline characteristics of study participants according to SII/SIRI quartile.

**Characteristics**	**Total *n* = 9,318**	**SII**					**SIRI**			
		**Q1**	**Q2**	**Q3**	**Q4**	***p***	**Q1**	**Q2**	**Q3**	**Q4**	***p***
		<**252.68**	**252.68–344.36**	**344.36–458.26**	> = **458.26**		<**0.428**	**0.428–0.607**	**0.607–0.872**	> = **0.872**	
		***n*** = **2,329**	***n*** = **2,330**	***n*** = **2,330**	***n*** = **2,329**		***n*** = **2,329**	***n*** = **2,330**	***n*** = **2,330**	***n*** = **2,329**	
Age (years)	57.89 (12.91)	59.33 (12.18)	58.18 (12.68)	56.72 (13.24)	57.33 (13.37)	< 0.001	57.71 (11.43)	57.59 (12.55)	57.94 (13.29)	58.32 (14.21)	0.029
BMI (kg/m^2^)	25.01 (3.58)	24.73 (3.57)	25.02 (3.53)	25.15 (3.53)	25.14 (3.67)	< 0.001	24.43 (3.39)	25.05 (3.60)	25.26 (3.61)	25.3 (3.66)	< 0.001
Male (%)	3,528 (37.86)	867 (37.23)	870 (37.34)	879 (37.73)	912 (39.16)	0.502	843 (36.20)	895 (38.41)	862 (37.00)	928 (39.85)	0.053
Married (%)	8,154 (87.51)	2,049 (87.98)	2,056 (88.24)	2,058 (88.33)	1,991 (85.49)	0.008	2,063 (88.58)	2,069 (88.80)	2,014 (86.44)	2,008 (86.22)	0.007
Urban (%)	5594 (60.03)	1,301 (55.86)	1,410 (60.52)	1,394 (59.83)	1,489 (63.93)	< 0.001	1,393 (59.81)	1,370 (58.80)	1,410 (60.52)	1,421 (61.01)	0.442
> = 9 years of education (%)	7,323 (78.59)	1,737 (74.58)	1,839 (78.93)	1,896 (81.37)	1,851 (79.48)	< 0.001	1,820 (78.15)	1,848 (79.31)	1,831 (78.58)	1,824 (78.32)	0.777
Current smoking (%)	1,548 (16.61)	325 (13.95)	357 (15.32)	404 (17.34)	462 (19.84)	< 0.001	154 (6.61)	292 (12.53)	424 (18.20)	678 (29.11)	< 0.001
Alcohol consumption (%)	1,120 (12.02)	274 (11.76)	288 (12.36)	262 (11.24)	296 (12.71)	0.428	170 (7.30)	257 (11.03)	302 (12.96)	391 (16.79)	< 0.001
Physical activity (%)	2,289 (24.57)	527 (22.63)	602 (25.84)	594 (25.49)	566 (24.3)	0.048	581 (24.95)	561 (24.08)	569 (24.42)	578 (24.82)	0.899
Hypertension (%)	3,806 (40.85)	905 (38.86)	884 (37.94)	947 (40.64)	1,070 (45.94)	< 0.001	820 (35.21)	903 (38.76)	1,001 (42.96)	1,082 (46.46)	< 0.001
Type 2 diabetes (%)	1,802 (19.34)	436 (18.72)	427 (18.33)	444 (19.06)	495 (21.25)	0.141	351 (15.07)	409 (17.55)	500 (21.46)	542 (23.27)	< 0.001
Dyslipidemia (%)	4,479 (48.07)	1,055 (45.30)	1,136 (48.76)	1,125 (48.28)	1,163 (49.94)	0.012	1,081 (46.41)	1,097 (47.08)	1,145 (49.14)	1,156 (49.64)	0.078
TG (mmol/L)	1.36 (0.95, 1.99)	1.29 (0.91, 1.88)	1.35 (0.95, 2.00)	1.40 (0.96, 2.06)	1.39 (0.98, 2.02)	< 0.001	1.26 (0.89, 1.87)	1.35 (0.94, 1.94)	1.39 (0.97, 2.06)	1.42 (1.00, 2.08)	< 0.001
TC (mmol/L)	5.49 (4.76, 6.23)	5.49 (4.78, 6.25)	5.56 (4.79, 6.27)	5.43 (4.74, 6.19)	5.46 (4.75, 6.23)	0.014	5.63 (4.92, 6.40)	5.5 (4.80, 6.24)	5.46 (4.69, 6.21)	5.35 (4.69, 6.06)	< 0.001
HDL (mmol/L)	1.33 (1.13, 1.57)	1.36 (1.16, 1.61)	1.33 (1.13, 1.58)	1.31 (1.13, 1.54)	1.32 (1.12, 1.54)	0.001	1.41 (1.21, 1.67)	1.35 (1.14, 1.58)	1.30 (1.11, 1.53)	1.27 (1.08, 1.48)	< 0.001
LDL (mmol/L)	3.07 (2.42, 3.75)	3.01 (2.36, 3.67)	3.07 (2.43, 3.79)	3.06 (2.41, 3.75)	3.13 (2.49, 3.78)	0.002	3.10 (2.47, 3.79)	3.07 (2.41, 3.79)	3.05 (2.40, 3.73)	3.04 (2.41, 3.68)	0.057
FPG (mmol/L)	5.47 (5.09, 6.09)	5.47 (5.09, 6.06)	5.47 (5.09, 6.06)	5.46 (5.08, 6.06)	5.50 (5.10, 6.17)	0.447	5.41 (5.07, 5.94)	5.47 (5.09, 6.04)	5.51 (5.10, 6.19)	5.53 (5.10, 6.23)	< 0.001
HbA1c (%)	5.40 (4.90, 6.00)	5.40 (4.90, 5.90)	5.40 (4.90, 6.00)	5.40 (4.90, 6.00)	5.40 (4.90, 6.00)	0.045	5.40 (4.90, 5.80)	5.40 (4.90, 5.90)	5.40 (4.90, 6.00)	5.40 (4.90, 6.10)	0.001
RBC (10^9^/L)	4.61 (4.36, 4.90)	4.57 (4.31, 4.85)	4.60 (4.36, 4.90)	4.62 (4.38, 4.91)	4.65 (4.39, 4.95)	< 0.001	4.50 (4.27, 4.75)	4.59 (4.36, 4.87)	4.63 (4.38, 4.93)	4.74 (4.46, 5.04)	< 0.001
WBC (10^9^/L)	6.18 (5.22, 7.28)	5.48 (4.67, 6.38)	5.94 (5.10, 6.86)	6.36 (5.49, 7.37)	7.12 (6.07, 8.30)	< 0.001	5.17 (4.49, 6.01)	5.88 (5.12, 6.68)	6.45 (5.63, 7.35)	7.50 (6.46, 8.68)	< 0.001
Platelets (10^9^/L)	199.00 (167.00, 234.00)	161.00 (133.00, 188.00)	192.00 (166.00, 217.00)	211.00 (184.00, 241.00)	236.00 (205.00, 270.00)	< 0.001	190.00 (159.00, 225.00)	197.00 (166.00, 231.00)	202.00 (169.00, 235.00)	208.00 (173.00, 246.00)	< 0.001
Neutrophils (10^9^/L)	3.54 (2.86, 4.39)	2.72 (2.28, 3.23)	3.29 (2.81, 3.82)	3.77 (3.23, 4.42)	4.64 (3.94, 5.48)	< 0.001	2.63 (2.24, 3.06)	3.27 (2.85, 3.73)	3.85 (3.35, 4.39)	4.84 (4.14, 5.66)	< 0.001
Lymphocyte (10^9^/L)	2.03 (1.66, 2.46)	2.24 (1.83, 2.69)	2.10 (1.74, 2.52)	2.01 (1.68, 2.40)	1.82 (1.49, 2.20)	< 0.001	2.11 (1.76, 2.57)	2.09 (1.72, 2.51)	2.01 (1.68, 2.41)	1.91 (1.54, 2.33)	< 0.001
Monocyte (10^9^/L)	0.35 (0.28, 0.44)	0.32 (0.26, 0.40)	0.34 (0.27, 0.42)	0.36 (0.29, 0.45)	0.39 (0.32, 0.50)	< 0.001	0.26 (0.22, 0.31)	0.33 (0.28, 0.38)	0.38 (0.33, 0.44)	0.48 (0.41, 0.57)	< 0.001

Data are mean (SD), *n* (%), or median (IQR). SII, systemic immune-inflammation index; SIRI, systemic inflammation response index; BMI, body mass index; TG, triglycerides; TC, total cholesterol; HDL, high-density lipoprotein cholesterol; LDL, low-density lipoprotein cholesterol; FPG, fasting plasma glucose; HbA1c, hemoglobin A1c; RBC, Red blood cell; WBC, White blood cell.

### Correlations of SII and SIRI with mortality

During a median follow-up period of 10.77 years (IQR: 10.68, 10.81), 967 deaths were documented. Cardiovascular disease accounted for the highest proportion of deaths (404 cases, 41.78%), followed by cancer (290 cases, 29.99%), respiratory disease (53 cases, 5.48%), and other causes (167 cases, 17.27%). Mortality rates exhibited a clear ascending gradient across increasing quartiles of both SII and SIRI. After multivariable adjustment, participants in the highest quartile (Q4) of SII had a significantly higher risk of all-cause mortality (HR = 1.35, 95% CI: 1.14–1.61), cardiovascular mortality (HR = 1.33, 95% CI: 1.02–1.73), and respiratory mortality (HR = 3.37, 95% CI: 1.44–7.90), but not cancer mortality. In comparison, participants in the highest quartile (Q4) of SIRI had a significantly higher risk of all-cause mortality (HR = 1.68, 95% CI: 1.39–2.04), cardiovascular mortality (HR = 1.40, 95% CI: 1.05–1.87), cancer mortality (HR = 1.45, 95% CI: 1.02–2.05), and respiratory mortality (HR = 3.07, 95% CI: 1.34–7.02). When SII and SIRI were modeled as log-transformed continuous variables, the results remained consistent: each unit increase in log-transformed SII or SIRI was significantly associated with elevated mortality risk, with effect directions and statistical significance aligning closely with quartile- based results (Table 2).

**Table 2:** Hazard ratios for all-cause mortality and cause-specific mortality in study participants according to SII/SIRI quartile.

**Variables**	**Person-years**	**Death cases**	**Mortality (1/1,000 person-years)**	**Crude HR (95% CI)**	***p***	Adjusted HR^*^(95% CI)	***p***
**All-cause mortality**							
**SII quartile**							
Q1	24,174	231	9.56	ref.		ref.	
Q2	24,290	222	9.14	0.95 (0.79–1.15)	0.620	1.04 (0.86–1.25)	0.698
Q3	24,275	209	8.61	0.90 (0.75–1.08)	0.263	1.04 (0.86–1.25)	0.703
Q4	23,843	305	12.79	1.35 (1.13–1.60)	0.001	1.35 (1.14–1.61)	0.001
Log SII	96,582	967	10.01	1.59 (1.16–2.16)	0.004	1.67 (1.24–2.25)	0.001
**SIRI quartile**	
Q1	24,486	156	6.37	ref.		ref.	
Q2	24,381	193	7.92	1.25 (1.01–1.54)	0.041	1.09 (0.89–1.35)	0.402
Q3	24,178	241	9.97	1.57 (1.29–1.92)	< 0.001	1.18 (0.96–1.45)	0.109
Q4	23,538	377	16.02	2.55 (2.12–3.08)	< 0.001	1.68 (1.39–2.04)	< 0.001
Log SIRI	96,582	967	10.01	5.41 (4.18–6.99)	< 0.001	2.83 (2.15–3.72)	< 0.001
**Cardiovascular mortality**							
**SII quartile**							
Q1	24,174	97	4.01	ref.		ref.	
Q2	24,290	97	3.99	0.99 (0.75–1.32)	0.960	1.08 (0.81–1.43)	0.599
Q3	24,275	80	3.30	0.82 (0.61–1.10)	0.186	0.93 (0.69–1.25)	0.629
Q4	23,843	130	5.45	1.37 (1.05–1.78)	0.020	1.33 (1.02–1.73)	0.036
Log SII	96,582	404	4.18	1.70 (1.05–2.76)	0.029	1.69 (1.07–2.66)	0.024
**SIRI quartile**	
Q1	24,486	70	2.86	ref.		ref.	
Q2	24,381	79	3.24	1.14 (0.82–1.57)	0.434	0.96 (0.70–1.33)	0.813
Q3	24,178	97	4.01	1.41 (1.04–1.92)	0.028	0.97 (0.71–1.32)	0.847
Q4	23,538	158	6.71	2.39 (1.80–3.17)	< 0.001	1.40 (1.05–1.87)	0.021
Log SIRI	96,582	404	4.18	5.27 (3.54–7.85)	< 0.001	2.27 (1.48–3.48)	< 0.001
**Cancer mortality**							
**SII quartile**							
Q1	24,174	73	3.02	ref.		ref.	
Q2	24,290	73	3.01	0.99 (0.72–1.37)	0.967	1.07 (0.77–1.48)	0.697
Q3	24,275	63	2.60	0.86 (0.61–1.2)	0.371	0.96 (0.69–1.35)	0.833
Q4	23,843	81	3.40	1.13 (0.82–1.55)	0.455	1.20 (0.87–1.65)	0.257
Log SII	96,582	290	3.00	1.03 (0.59–1.81)	0.918	1.21 (0.69–2.09)	0.507
**SIRI quartile**	
Q1	24,486	51	2.08	ref.		ref.	
Q2	24,381	71	2.91	1.40 (0.98–2.01)	0.066	1.27 (0.89–1.82)	0.195
Q3	24,178	72	2.98	1.44 (1.00–2.06)	0.048	1.19 (0.83–1.70)	0.357
Q4	23,538	96	4.08	1.98 (1.41–2.78)	< 0.001	1.45 (1.02–2.05)	0.039
Log SIRI	96,582	290	3.00	3.66 (2.27–5.88)	< 0.001	2.25 (1.36–3.70)	0.001
**Respiratory mortality**							
**SII quartile**							
Q1	24,174	7	0.29	ref.		ref.	
Q2	24,290	9	0.37	1.28 (0.48–3.43)	0.627	1.38 (0.51–3.70)	0.525
Q3	24,275	15	0.62	2.13 (0.87–5.22)	0.099	2.48 (1.01–6.09)	0.047
Q4	23,843	22	0.92	3.19 (1.36–7.48)	0.007	3.37 (1.44–7.90)	0.005
Log SII	96,582	53	0.55	9.98 (2.87–34.70)	< 0.001	9.72 (2.89–32.75)	< 0.001
**SIRI quartile**	
Q1	24,486	7	0.29	ref.		ref.	
Q2	24,381	2	0.08	0.29 (0.06–1.38)	0.120	0.25 (0.05–1.21)	0.085
Q3	24,178	14	0.58	2.04 (0.82–5.04)	0.125	1.56 (0.63–3.88)	0.337
Q4	23,538	30	1.27	4.51 (1.98–10.27)	< 0.001	3.07 (1.34–7.02)	0.008
Log SIRI	96,582	53	0.55	20.36 (7.17–57.83)	< 0.001	12.84 (4.28–38.53)	< 0.001

^*^Models for all–cause, cardiovascular, and cancer mortality were adjusted for age, sex, residential area, marriage status, education, current smoking, alcohol consumption, physical activity, body mass index, hypertension, type 2 diabetes, and dyslipidemia. The model for respiratory mortality was adjusted for age and sex. SII, systemic immune-inflammation index; SIRI, systemic inflammation response index.

### Dose-response relationship between SII, SIRI and mortality

**Figure 1:**
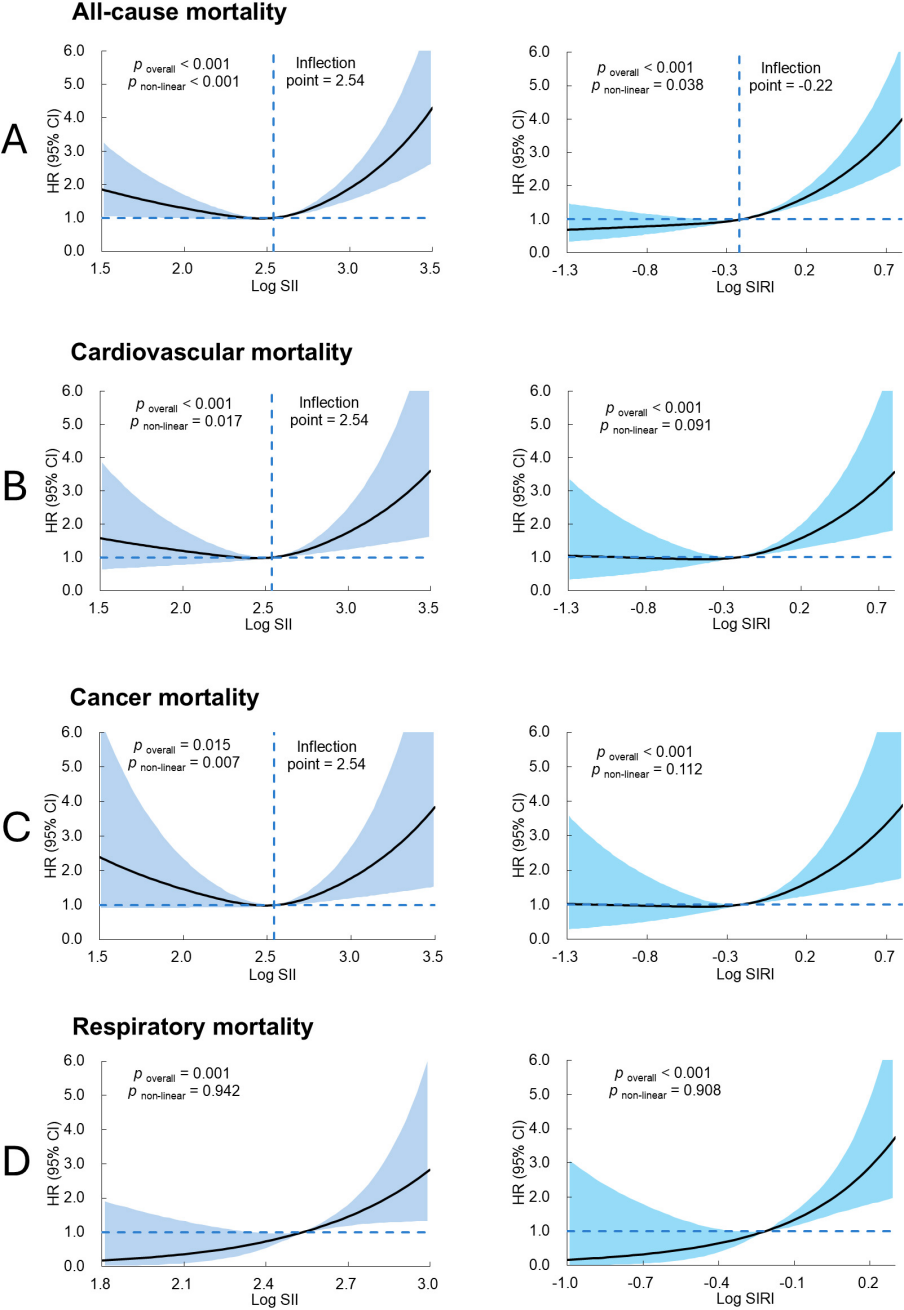
Multivariable adjusted spline curves for association between SII, SIRI and risk for **(A)** all-cause mortality, **(B)** cardiovascular mortality, **(C)** cancer mortality, and **(D)** respiratory mortality. Models for all-cause, cardiovascular, and cancer mortality were adjusted for age, sex, residential area, marriage status, education, current smoking, alcohol consumption, physical activity, body mass index, hypertension, type 2 diabetes, and dyslipidemia. The model for respiratory mortality was adjusted for age and sex. Knots were set at the 5th, 50th, and 95th percentile. Reference was set at the median level of log SII (2.54) or log SIRI (−0.22).

The dose-response relationships between log-transformed SII, SIRI and the risks of all-cause and cause-specific mortality are presented in Figure 1. Restricted cubic spline analysis revealed that elevated SII and SIRI were associated with increased risks of all-cause, cardiovascular, cancer, and respiratory mortality. Specifically, SII exhibited a U-shaped association with all-cause mortality and nonlinear associations with cardiovascular and cancer mortality. In contrast, SIRI demonstrated a J-shaped association with all-cause mortality, while no nonlinear associations were detected between SIRI and cardiovascular or cancer mortality. Linear associations were observed for both SII and SIRI with respiratory mortality.

### Subgroup analyses

Table 3 presents the results of subgroup analyses for the associations of SII/SIRI with all-cause mortality. Significant effect modification was observed by age for SIRI (*p* for interaction < 0.001). The association was markedly stronger in participants aged < 60 years (Q4 vs. Q1: HR = 2.42, 95% CI: 1.36–4.33) compared to those aged ≥60 years (Q4 vs. Q1: HR = 1.57, 95% CI: 1.28–1.92). Similar trend was noted for SII, though not statistically significant for interaction (*p* for interaction = 0.219). The positive associations remained consistent across sex, BMI, hypertension, and type 2 diabetes status (all *p* for interaction >0.05). Notably, higher mortality risks associated with the highest quartiles of SII and SIRI were particularly pronounced in subgroups such as non-obese individuals and those without hypertension. No statistically significant interactions were found for the associations of SII/SIRI with cardiovascular, cancer, or respiratory mortality across any of the examined subgroups (all *p* for interaction >0.05).

**Table 3:** Subgroup analysis of hazard ratios for all-cause mortality in study participants according to SII/SIRI quartile.

**Variables**	**SII**			**SIRI**		
	**Adjusted HR** ^*^ **(95% CI)**	***p***	***p*** **for interaction**	**Adjusted HR** ^*^ **(95% CI)**	***p***	***p*** **for interaction**
Age (years)			0.219			< 0.001
<**60**	
Q1	ref.			ref.		
Q2	0.86 (0.46–1.60)	0.632		1.72 (0.95–3.10)	0.072	
Q3	1.39 (0.80–2.43)	0.241		2.00 (1.11–3.59)	0.020	
Q4	2.19 (1.31–3.66)	0.003		2.42 (1.36–4.33)	0.003	
> = **60**	
Q1	ref.			ref.		
Q2	1.06 (0.87–1.28)	0.584		1.01 (0.81–1.27)	0.917	
Q3	0.99 (0.81–1.21)	0.900		1.08 (0.87–1.34)	0.470	
Q4	1.27 (1.06–1.53)	0.010		1.57 (1.28–1.92)	< 0.001	
Sex			0.374			0.717
**Male**	
Q1	ref.			ref.		
Q2	1.04 (0.77–1.40)	0.801		1.00 (0.72–1.38)	0.995	
Q3	1.17 (0.87–1.57)	0.295		0.89 (0.64–1.23)	0.473	
Q4	1.33 (1.00–1.75)	0.046		1.45 (1.08–1.94)	0.013	
**Female**	
Q1	ref.			ref.		
Q2	1.03 (0.81–1.30)	0.815		1.16 (0.88–1.53)	0.294	
Q3	0.95 (0.74–1.21)	0.670		1.41 (1.09–1.83)	0.010	
Q4	1.37 (1.10–1.70)	0.005		1.84 (1.43–2.37)	< 0.001	
BMI (kg/m^2^)			0.692			0.425
>= **24**	
Q1	ref.		ref.		
Q2	1.02 (0.79–1.31)	0.905		0.98 (0.73–1.33)	0.919	
Q3	1.06 (0.82–1.38)	0.637		1.10 (0.82–1.47)	0.527	
Q4	1.25 (0.98–1.60)	0.075		1.48 (1.12–1.94)	0.006	
<**24**	
Q1	ref.			ref.		
Q2	1.05 (0.80–1.37)	0.717		1.21 (0.90–1.62)	0.216	
Q3	1.03 (0.78–1.35)	0.859		1.28 (0.96–1.70)	0.091	
Q4	1.50 (1.18–1.91)	0.001		1.96 (1.50–2.56)	< 0.001	
Hypertension			0.348			0.225
**Yes**	
Q1	ref.			ref.		
Q2	0.94 (0.74–1.21)	0.648		1.07 (0.82–1.41)	0.606	
Q3	1.00 (0.79–1.27)	0.999		1.04 (0.80–1.36)	0.759	
Q4	1.31 (1.05–1.63)	0.017		1.44 (1.13–1.85)	0.004	
**No**	
Q1	ref.			ref.		
Q2	1.17 (0.88–1.54)	0.272		1.08 (0.77–1.50)	0.665	
Q3	1.07 (0.80–1.45)	0.634		1.35 (0.98–1.84)	0.063	
Q4	1.41 (1.07–1.86)	0.014		2.01 (1.49–2.71)	< 0.001	
Type 2 diabetes			0.233			0.353
**Yes**	
Q1	ref.			ref.		
Q2	0.93 (0.68–1.29)	0.674		0.79 (0.54–1.14)	0.205	
Q3	1.10 (0.81–1.50)	0.527		1.10 (0.79–1.52)	0.578	
Q4	1.48 (1.13–1.95)	0.005		1.51 (1.11–2.06)	0.009	
**No**	
Q1	ref.			ref.		
Q2	1.07 (0.85–1.34)	0.549		1.27 (0.98–1.65)	0.067	
Q3	0.98 (0.77–1.24)	0.869		1.20 (0.93–1.56)	0.163	
Q4	1.27 (1.01–1.58)	0.037		1.75 (1.37–2.22)	< 0.001	

^*^Adjusted for age, sex, residential area, marriage status, education, current smoking, alcohol consumption, physical activity, body mass index, hypertension, type 2 diabetes, and dyslipidemia, excluding the stratification factor. SII, systemic immune–inflammation index; SIRI, systemic inflammation response index; BMI, body mass index.

### The predictive performance of SII and SIRI for mortality

Table 4 demonstrates that the addition of either the SII or SIRI significantly enhanced the predictive performance beyond the basic model incorporating conventional risk factors for all-cause mortality and other mortality. The C-index (95% CI) of the basic predicting models constructed with conventional risk factors were 0.837 (0.825–0.849) for all-cause mortality, 0.894 (0.826–0.904) for CVD-related mortality, and 0.766 (0.751–0.904) for cancer-related mortality, respectively. For all-cause mortality prediction, adding SIRI significantly improved the C-index, IDI, and NRI of the basic model, whereas SII significantly increased the C-index and NRI. In predicting cardiovascular mortality, SIRI significantly improved the IDI, while SII provided little improvement. For cancer mortality prediction, the inclusion of SIRI significantly increased the C-index and IDI, whereas SII showed little improvement.

**Table 4:** Improvement in predicting all-cause mortality and cause-specific mortality by adding SII/SIRI to basic model.

**Models**	**C-index (95% CI)**	**Change in C-index (95% CI)**	**IDI, % (95% CI)**	**NRI, % (95% CI)**
**All-cause mortality**	
Basic model	0.837 (0.825–0.849)	ref.	ref.	ref.
Plus SII	0.838 (0.827–0.851)	0.001 (0.001–0.002)^*^	0.157 (−0.058 to 0.376)	8.432 (0.285–16.354)^*^
Plus SIRI	0.842 (0.831–0.853)	0.005 (0.003–0.007)^*^	0.611 (0.092–1.086)^*^	9.408 (1.418–16.209)^*^
**Cardiovascular mortality**	
Basic model	0.894 (0.826–0.904)	ref.	ref.	ref.
Plus SII	0.895 (0.827–0.905)	0.001 (0.000–0.002)	0.178 (−0.127 to 0.386)	5.632 (−1.582 to 16.469)
Plus SIRI	0.896 (0.831–0.906)	0.002 (0.001–0.007)	0.601 (0.042–1.120)^*^	1.836 (−6.205 to 24.868)
**Cancer mortality**	
Basic model	0.766 (0.751–0.904)	ref.	ref.	ref.
Plus SII	0.767 (0.752–0.905)	0.001 (0.000–0.002)	0.089 (−0.132 to 0.364)	1.719 (−5.681 to 14.698)
Plus SIRI	0.772 (0.756–0.906)	0.007 (0.000–0.010)^*^	0.599 (−0.039 to 1.082)^*^	5.98 (−5.129 to 24.553)

^*^p < 0.05. Basic model included age, sex, residential area, marriage status, education, current smoking, alcohol consumption, physical activity, body mass index, hypertension, type 2 diabetes, and dyslipidemia. IDI, integrated discrimination improvement; NRI, net reclassification improvement, SII: systemic immune-inflammation index, SIRI: systemic inflammation response index.

## Discussion

In the present community-based cohort study, we identified that both elevated SII and SIRI are significantly associated with increased risks of all-cause, cardiovascular, and respiratory mortality in a community-based cohort, with SIRI showing an additional significant association with cancer mortality. Significant dose-response relationships were observed for both SII and SIRI with all-cause and cause-specific mortality. The associations were robust across most subgroups, though particularly pronounced for SIRI among younger participants. Incorporating these indices, especially SIRI, into conventional risk models consistently improved the predictive performance for mortality, underscoring their potential clinical utility as integrative biomarkers of systemic inflammation for mortality risk stratification. The results of this study have practical implications for informing and refining health promotion strategies within Chinese communities. Their generalizability to other demographic and geographic settings warrants investigation in future studies.

The results of this study corroborate existing evidence that investigating the predictive value of SII and SIRI for all-cause and cause-specific mortality. As systemic inflammatory biomarkers, SII and SIRI have been linked to increased mortality in both the general population and patients with various diseases. In the general population, individuals with higher levels of SII and SIRI are associated with elevated risks of all-cause and cardiovascular mortality (15, 16). Similar associations have been observed in patients with conditions such as diabetes, asthma, sarcopenia, osteoarthritis, and rheumatoid arthritis (17–21). The relationship between SII and SIRI and respiratory mortality remains underexplored in previous research. Existing evidence indicates that elevated SII and SIRI levels are associated with adverse respiratory outcomes. Li, et al. suggest that patients with elevated SII and SIRI levels are more susceptible to acute exacerbation and readmission events due to chronic obstructive pulmonary disease (COPD) within 1 year after hospital discharge (22). Similarly, an observational study with 2-years follow-up suggests that elevated SII is associated with increased risks of short-term and long-term adverse outcomes among inpatients with acute exacerbations of COPD (23). Individuals with higher SII levels are linked to higher prevalence of COPD, COPD patients with a higher SII levels have a higher risk of all-cause mortality (24). In the present study, we observed notable associations between elevated SII and SIRI and an increased risk of respiratory mortality. However, given the limited number of respiratory death events, the findings should be interpreted with caution as they may be statistically unstable. Further validation is needed before any causal implications can be drawn. If confirmed, such associations could offer clinicians additional perspectives for assessing systemic inflammation and respiratory health.

Both SIRI and SII are robust systemic inflammatory markers, and clinically practical due to their derivation from routine blood counts. Although initial evidence suggests that both can predict responses to anti-inflammatory therapy, their applicability in the general population necessitates further investigation (25). Data from NHANES showed that SIRI and SII are independent risk factors for all-cause and cardiovascular mortality in obese individuals, and the predictive performance of SIRI for both outcomes significantly surpassed that of SII (26). In the asthmatic population, the predictive value of SIRI for stroke prevalence was superior to that of SII (27). In patients with metabolic dysfunction-associated steatotic liver disease, SIRI also outperformed SII in predicting cardiovascular mortality risk, establishing it as a useful tool for mortality risk stratification (28). Both SIRI and SII incorporate neutrophil and lymphocyte counts, but they are distinguished by the inclusion of different additional cell types. SIRI integrates monocyte counts, whereas SII focuses on platelet counts. Monocytes play a crucial role in the pathogenesis of atherosclerosis, directly participating in processes such as foam cell formation, cytokine and chemokine secretion, and plaque destabilization (29, 30). Beyond their role in cardiovascular disease, monocytes also serve as early biomarkers for a spectrum of chronic diseases, such as obesity, chronic obstructive pulmonary disease, lung fibrosis, and lung cancer (31). Accordingly, SIRI more specifically captures monocyte-mediated inflammatory responses. Elevated SIRI reflects monocyte HLA-DR downregulation and impaired antigen presentation, driving tumor immune escape and poorer cancer prognosis—linking it closely to adaptive immunity and tumor surveillance 31191529 25999436. In contrast, SII integrates neutrophil- and platelet-driven pathways central to thrombo-inflammation, thereby reflecting immune activation and vascular injury 30228378. These mechanistic distinctions underpin the differential prognostic performance of the two indices.

The superior predictive performance of SIRI over SII for cancer mortality may be attributed to its alignment with tumor-specific immunobiology. SIRI directly incorporates monocytes, the precursors to tumor-associated macrophages, which are pivotal in promoting tumor progression, angiogenesis, and immune suppression (32). In contrast, SII includes platelets, which are more variably involved in thrombo-inflammatory pathways. Furthermore, by using lymphocytes as the denominator, SIRI is exquisitely sensitive to lymphopenia—a hallmark of systemic immune exhaustion that is strongly linked to poor cancer prognosis (33). Therefore, SIRI reflects both pro-tumor inflammation and impaired immune surveillance, explaining its superior ability to predict cancer mortality.

Notably, a significant interaction with age was observed for the association between SIRI and all-cause mortality, using 60 years as the cutoff. Age independently modified the effect of SIRI on all-cause mortality risk. Consistent with our findings, Jin et al. demonstrated that the association between elevated SIRI and increased MI incidence was modified by age and remained significant only in participants aged < 60 (16). Furthermore, both the SII and SIRI have been identified as independent risk factors for coronary heart disease in young adults under 45 years of age (34). These observations outline an age-specific pattern: In young and middle-aged adults, inflammation markers often serve as early and relatively specific indicators of a deviation from health homeostasis; in older adults, inflammation has become a physiological baseline, accompanied by multiple comorbidities and geriatric syndromes (35–37). Thus, the predictive specificity of inflammatory marker is substantially diminished in the elderly population. These differential effects across age groups underscore the value of SIRI as an age-specific biomarker.

This study has several strengths, including a representative cohort recruited through probability sampling from community residents, systematically implemented measurements, adjustment for a wide range of potential confounders, and linkage to a reliable mortality surveillance system. However, some limitations should be acknowledged. First, the follow-up period could be extended to establish more robust associations. Second, SII and SIRI were assessed only at baseline, which may not fully reflect long-term exposure levels due to biological variability, potentially leading to exposure misclassification and attenuation of the observed associations. Third, despite adjustment for numerous covariates, data on several potentially influential factors—such as dietary habits, medication use, comorbidities, socioeconomic status, and environmental exposures—were not available, leaving the possibility of residual confounding. Fourth, the numerous statistical tests conducted increase the possibility of chance findings, and that the subgroup results in particular should be interpreted with caution and require future validation.

## Conclusions

In conclusion, we suggest that SII and SIRI are independent predictors of all-cause, cardiovascular, and respiratory mortality, with SIRI additionally linked to cancer mortality. Adding these biomarkers yielded modest improvements in conventional model performance. Our results highlight systemic inflammation as a relevant biological pathway and public health target. Further validation is needed before any risk stratification role can be established.

## Data Availability

The raw data supporting the conclusions of this article will be made available by the authors, without undue reservation.
