# Supplementary material for: Impact of systemic immune-inflammation index and systemic inflammation response index on all-cause and cause-specific mortality: a community-based cohort study
Source: Front Med (Lausanne). 2026 Mar 25;13:1784058. doi: 10.3389/fmed.2026.1784058 (PMC13057441; doi:10.3389/fmed.2026.1784058)
Supplement: Supplementary file 1 [file Supplementary_file_1.docx]

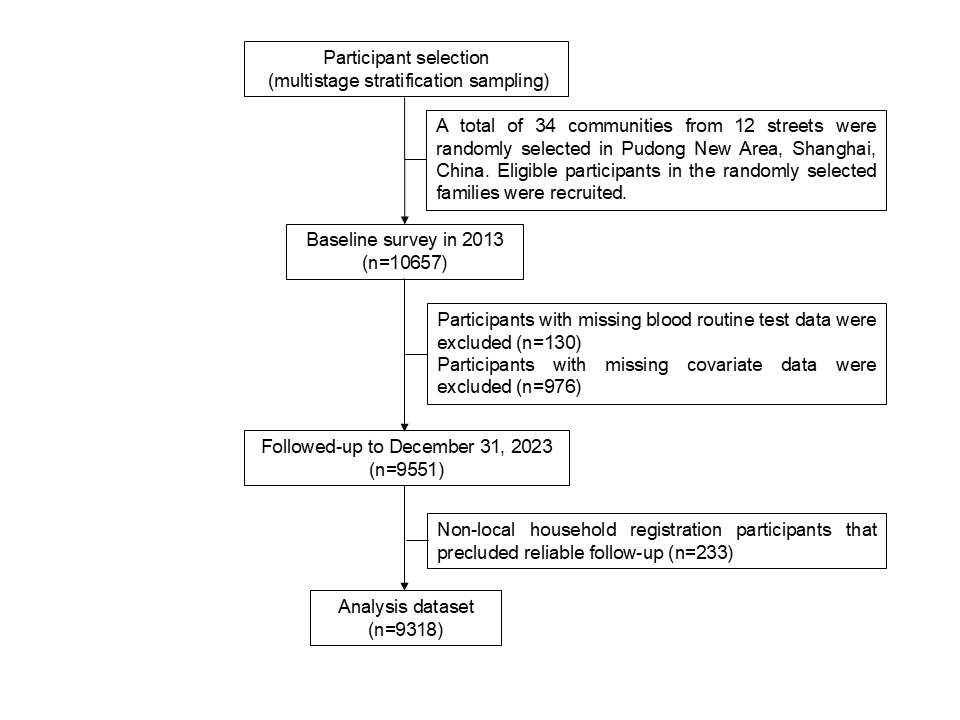


Supplemental Figure 1. Diagram of this study.

Supplemental Table 1. Test of proportional hazards assumption by SII/SIRI quartile

| Variables | SII quartile | | SIRI quartile | |
| --- | --- | --- | --- | --- |
|  | χ^2^ | *p* | χ^2^ | *p* |
| **All-cause mortality** |  |  |  |  |
| Age | 2.003 | 0.157 | 2.474 | 0.116 |
| Marriage status | 1.460 | 0.227 | 1.487 | 0.223 |
| Current smoking | 0.434 | 0.510 | 1.197 | 0.274 |
| Type 2 diabetes | 1.399 | 0.237 | 1.798 | 0.180 |
| Global | 5.103 | 0.647 | 11.720 | 0.110 |
| **Cardiovascular mortality** |  |  |  |  |
| Age | 0.256 | 0.613 | 0.427 | 0.514 |
| Marriage status | 1.604 | 0.205 | 1.608 | 0.205 |
| Current smoking | 1.640 | 0.200 | 2.180 | 0.140 |
| Hypertension | 0.028 | 0.868 | 0.027 | 0.869 |
| Type 2 diabetes | 1.760 | 0.185 | 2.048 | 0.152 |
| Global | 5.601 | 0.347 | 7.788 | 0.455 |
| **Cancer mortality** |  |  |  |  |
| Age | 0.040 | 0.842 | 0.040 | 0.842 |
| Residential area | 0.301 | 0.583 | 0.301 | 0.583 |
| Current smoking | 0.069 | 0.792 | 0.069 | 0.792 |
| Global | 0.395 | 0.941 | 0.395 | 0.941 |
| **Respiratory mortality** |  |  |  |  |
| Age | 0.148 | 0.701 | 0.252 | 0.616 |
| Global | 3.357 | 0.500 | 2.612 | 0.625 |
